# Crosstalk Among circRNA/lncRNA, miRNA, and mRNA in Osteoarthritis

**DOI:** 10.3389/fcell.2021.774370

**Published:** 2021-12-15

**Authors:** Hui Kong, Ming-Li Sun, Xin-An Zhang, Xue-Qiang Wang

**Affiliations:** ^1^ College of Kinesiology, Shenyang Sport University, Shenyang, China; ^2^ Department of Sport Rehabilitation, Shanghai University of Sport, Shanghai, China; ^3^ Department of Rehabilitation Medicine, Shanghai Shangti Orthopaedic Hospital, Shanghai, China

**Keywords:** osteoarthritis, miRNA, lncRNA, circRNA, lncRNA/circRNA-miRNA-mRNA axis

## Abstract

Osteoarthritis (OA) is a joint disease that is pervasive in life, and the incidence and mortality of OA are increasing, causing many adverse effects on people’s life. Therefore, it is very vital to identify new biomarkers and therapeutic targets in the clinical diagnosis and treatment of OA. ncRNA is a nonprotein-coding RNA that does not translate into proteins but participates in protein translation. At the RNA level, it can perform biological functions. Many studies have found that miRNA, lncRNA, and circRNA are closely related to the course of OA and play important regulatory roles in transcription, post-transcription, and post-translation, which can be used as biological targets for the prevention, diagnosis, and treatment of OA. In this review, we summarized and described the various roles of different types of miRNA, lncRNA, and circRNA in OA, the roles of different lncRNA/circRNA-miRNA-mRNA axis in OA, and the possible prospects of these ncRNAs in clinical application.

## Introduction

Osteoarthritis (OA) is a joint disease that is pervasive in life. It is largely caused by cartilaginous injury and affects the whole joint tissue (Pereira et al., 2015). Nearly half of people over 65 suffer from OA.(Sakalauskienė and Jauniškienė, 2010; Glyn-Jones et al., 2015). Globally, the incidence and mortality of OA are increasing (Bijlsma et al., 2011). Arthrodynia, swelling, and inability to move freely are the main symptoms of OA and cause many adverse effects on people’s lives. Several risk factors (Prieto-Alhambra et al., 2014), including age, sex, obesity, genetics, and joint damage, have been linked to OA progression (Felson et al., 2000; Vincent, 2019; Abramoff and Caldera, 2020). Articular cartilage degeneration and secondary osteogenesis are the main pathological manifestations of OA (Burr and Gallant, 2012). The long-term development of OA will not only affect people’s behaviors and activities but also cause depression, anxiety, and other negative emotions (Litwic et al., 2013). To provide more perfect, targeted treatment for patients with OA, the progression of OA needs to be studied. The specific pathogenesis of OA may be related to metalloproteinases (Mehana et al., 2019), cytokines (Boehme and Rolauffs, 2018), signaling pathways (Rigoglou and Papavassiliou, 2013), and noncoding RNA (ncRNA) (Sondag and Haqqi, 2016).

ncRNA is a nonprotein-coding RNA that does not translate into proteins but participates in protein translation. At the RNA level, it can perform biological functions (Wu et al., 2019). microRNA (miRNA), long ncRNA (lncRNA), circular RNA (circRNA), ribosomal RNA (rRNA), transfer RNA (tRNA), small nuclear RNA (snRNA), small nucleolar RNA (snoRNA), small interfering RNA (siRNA), short hairpin RNA (shRNA) and Piwi-interactingRNA (piRNA) are the main ncRNAs(Chen et al., 2021). Studies have found that ncRNA is closely related to the occurrence of several diseases for the past few years (Esteller, 2011; Wang et al., 2019b). For example, promoter CpG methylation of two genes encoding members of the miR-200 family can easily lead to the occurrence and development of breast and colorectal cancer (Lim et al., 2013); miR-34b/c is a critical tumor suppressor. The methylation of miR-34b/c CpG island leads to the silence of miR-34b/c, thus increasing the incidence of tumors (Toyota et al., 2008); the decreased expression of miR-133 may induce myocardial hypertrophy by targeting the beta-1 adrenergic receptor pathway (Castaldi et al., 2014). Many studies have also found that miRNA, lncRNA, and circRNA are closely related to the course of OA, and play important regulatory roles in transcription, post-transcription, and post-translation (Li et al., 2019b; Zhang et al., 2021e). The interaction between lncRNA/circRNA, miRNA, and mRNA has attracted increasing attention. For example, lncRNA/circRNA can bind to miRNA, reduce the inhibitory effect of miRNA on mRNA, participate in regulating the progress of chondrocyte proliferation and apoptosis, extracellular matrix (ECM) degradation and inflammatory response in the progress of OA. Furthermore, lncRNA-p21 could induce chondrocyte apoptosis and slow the process of OA by binding to miR-451 and promoting the expression of downstream target gene mRNA (Tang et al., 2018a). This review describes the roles of miRNA, lncRNA, and circRNA in OA and the role of the lncRNA/circRNA–miRNA–mRNA axis in OA.

## miRNAs and OA

**FIGURE 1:**
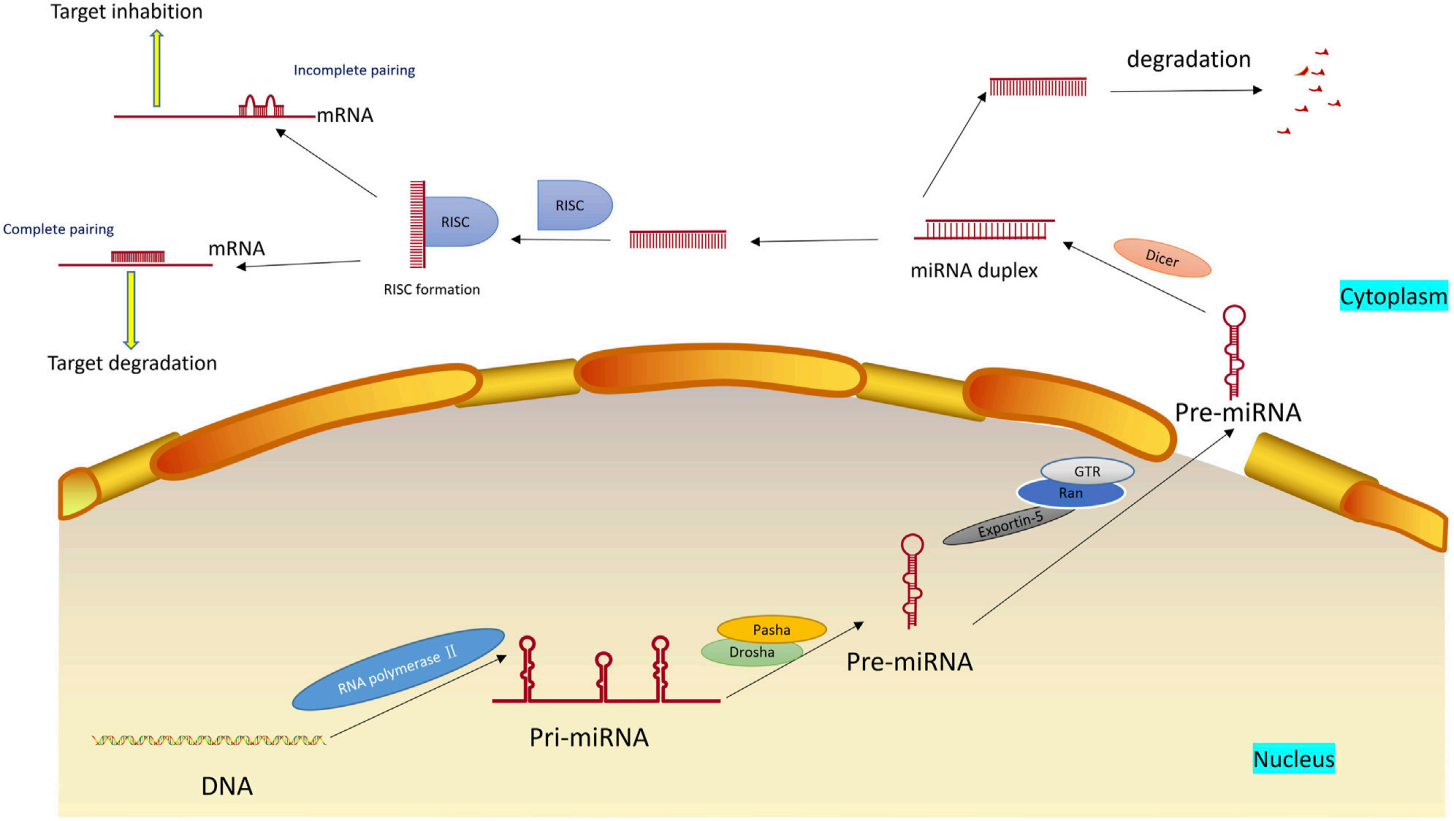
Formation and mechanism of miRNA. miRNAs are first transcripted into longer primary miRNAs in the nucleus and then processed into hairpin RNAs of 60–70 nucleotides in the nucleus by Drosha, Pasha et al. The precursor miRNAs are transported out of the cell nucleus with the help of the Ran-GTP-dependent nucleoplasmic/cell transporter Exprotin-5 and split into 21–25 nucleotide length double-stranded miRNAs in the cytoplasm by Dicer. Subsequently, the double helix is derotated by the action of the derotation enzyme, and one of the strands is integrated into the RNA-induced silencing complex (RISC), an asymmetric RISC assembly is formed, and the other chain is immediately degraded.

miRNA is a single-stranded RNA molecule with a length of about 20–24 nucleotides (Correia de Sousa et al., 2019). It belongs to one type of ncRNA and widely exists in eukaryotes to regulate the expression of other genes. miRNA regulates gene expression based on complete or incomplete pairing with mRNA. In most cases, the single-stranded miRNA in the complex is paired with the 3′UTR of the target mRNA in an incomplete complementary manner, blocking the translation of the gene and regulating gene expression. This process, called translation inhibition, is mainly found in animal cells. When the miRNA is completely complementary to the 3′UTR of the target mRNA, the mRNA in the complementary region would be specifically broken, eventually leading to gene silencing, and the process called post-transcriptional gene silencing, which will eventually lead to the degradation of target mRNA, mainly exists in plant cells (Liu et al., 2014a). The same gene can be regulated by multiple miRNAs, and multiple target genes can be regulated by the same miRNA (Iacona and Lutz, 2019). The formation and mechanism of miRNA are shown in Figure 1.

With the deepening of research, miRNAs have been discovered and studied increasingly, and they have become a potential target in disease prevention and treatment. miRNA has many functions roles in human diseases, such as regulating cell autophagy (Li et al., 2019h), epigenesis (Yao et al., 2019), glucose metabolism (Fu et al., 2015). Chen et al. (2018c) developed a computational model for disease association prediction to detect potential miRNA-disease associations accurately and efficiently. By studying three common human cancers (Zhang et al., 2021b), namely, colon cancer, esophagus cancer, and kidney cancer, many miRNAs were confirmed to be connected with the three kinds of cancer. In addition, many studies have proven that miRNA is related to the pathological processes of intervertebral disc degeneration (Shi et al., 2021b), muscle atrophy (Zhang et al., 2021a), and cardiovascular diseases (Liu et al., 2021a).

Currently, growing findings reveal that miRNA expression level changes exist in various tissues of patients with OA, leading to abnormal target gene expression. miRNA has many functions in OA, such as regulating cell autophagy and apoptosis (Yu et al., 2019b), inflammatory reaction (Sui et al., 2019), and cartilage degradation (Guo et al., 2020). Changes in miRNA expression levels in different tissues can be experimented with by gene sequencing. Gene sequencing is a new type of gene detection technology, which can analyze and determine the whole sequence of genes from blood or saliva to predict the possibility of suffering from various diseases and lock in individual diseased genes for early prevention and treatment. Zhou et al. (2020b) revealed 21 differentially expressed miRNAs in synovial tissues from OA patients compared with normal controls by gene sequencing technology. The expression levels of the first two DEmiRNAs(hsa-miR-17-5p and hsa-miR-20b-5p), which cover most of the DEmRNAs, were analyzed and found to be down-regulated in OA, which was also confirmed by qRT-PCR verification. Ntoumou et al. (2017) assessed differential miRNA expression by microarray analysis in the serum of patients with OA. Compared with the control group, 279 miRNAs were differentially expressed in OA. This study focused on analyzing and studying three differentially expressed miRNAs: hsa-miR-140-3p, hsa-miR-671-3p, and hsa-miR-33b-3p. We found that the expression of these three miRNAs was down-regulated in the serum of OA patients. Through serum microRNA array analysis and bioinformatics analysis, they determined that these three miRNAs were potential OA biomarkers involved in the metabolic processes of insulin and cholesterol. OA is a metabolic disease, and insulin resistance plays a vital role in metabolic syndrome. Therefore, the metabolic processes of insulin and cholesterol in the body are closely related to OA. In addition, based on RNA sequencing and miRNA analysis, Wu et al. (2021a) identified that miR-210-5p is highly enriched in the exosomes of OA sclerotic subchondral osteoblasts, triggering the expression of genes associated with catabolism in articular chondrocytes. Therefore, the abnormal up-regulation of miR-210-5p in exosomes could serve as a marker for OA. Notably, miRNA show obvious tissue specificity in different OA tissues. For example, the expression of miR-125b-5p in synovial fluid and chondrocytes is different in OA patients. Ge et al. (2017) found by PCR that miR-125b-5p in synovial fluid was significantly up-regulated in OA patients compared with normal subjects, promoting synovial cell apoptosis by targeting syvn1. Rasheed et al. (2019) treated chondrocytes with IL-1β to construct OA cell models and determined the expression of miR-125b-5p using Taqman analysis. They found that miR-125b-5p in chondrocytes was significantly down-regulated compared to healthy individuals and regulated inflammatory genes in OA chondrocytes by targeting TRAF6. Our appeal study found that expression levels of multiple miRNAs in the synovial membrane, cartilage, and subchondral bone were altered in OA patients compared to healthy individuals. In addition, even in the same tissue, if in different stages of development, the expression of miRNAs may be different. For example, in different stages of the knee joint cartilage of rats, Sun et al. (2011) used Solexa sequencing and RT-qPCR detection for the expression of miRNAs. They tested the miRNAs in the rat knee joint cartilage at the starting point, on Day 21 and Day 42, and found that the expression of miRNAs was different at each stage. Among them, 4 representative miRNAs were selected for further analysis. Compared with the initial stage, the expressions of aggrecan, colia1, and ColXa1 were up-regulated on day 21. The expression of ColXa1 was up-regulated on day 42, whereas those of aggrecan and colia1 were down-regulated. The expression of Sox9 showed minimal change during the three stages. Gabler et al. (2015) found that miRNA could control the differentiation of chondrocytes and regulate the occurrence of OA. During the development of human bone marrow mesenchymal stem cells (HMSCs), the expression of miRNA in different development stages is also different. By microarray analysis, the miR spectra of HMSCs in patients with OA at different development time points were measured. Among the 1,349 detected miRNAs, 553 were expressed in cartilage formation, they further performed miRNAs detection at 7, 14, 21, and 42 days after cartilage formation and found that their expression of miRNAs was also different. In summary, the expression of miRNAs in OA patients is different in different tissues and between different stages of development of the same tissue.

It is well known that many intracellular signaling pathways, such as nuclear factor-kappaB(NF-κB) and transforming growth factor *β* (TGF-β) played an vital roles in the pathogenesis of OA (Nishimura et al., 2020). In recent years, more studies discovered that miRNA can delay the pathological process of OA by promoting or inhibiting these pathways (Xu et al., 2016). NF-κB is an essential nuclear transcription factor in cells participating in the inflammatory and immune response of the body and apoptosis regulation (Lawrence, 2009). For example, as the 3′UTR of NF-κB contains the binding site of miR-143 and miR-124, when the DNA methylation degree of miR-143 and miR-124 promoters is reduced, the expression of miR-143 and miR-124 is up-regulated, and the transcription process is activated, thereby inhibiting the NF-κB signaling pathway, inhibiting apoptosis and delaying the progression of OA (Qiu et al., 2020). Similarly, When the expression levels of miR-34a and miR-181a were decreased, the expression of the BCL2 gene was increased, thereby limiting the term of NF-κ B translocation into the nucleus in OA Chondrocytes cultures and eventually reducing apoptosis and oxidative stressl (Cheleschi et al., 2019). The TGF-β signaling pathway is involved in many cellular processes in mature organisms and developing embryos, including cell growth, differentiation, apoptosis, dynamic cell balance, and other cellular functions. By promoting or inhibiting the TGF-β signaling pathway, we can regulate the cellular processes, thereby inducing or delaying the progression of OA (Shen et al., 2014). Hu et al. (2019b) established OA mouse models. QPCR and Western blot were used to compare the expression of miR-455-3p and PAK2 in the cartilage of healthy individuals and patients with OA, and the luciferase reporter gene was used to analyze the interaction between them. The results showed that miR-455-3p could inhibit the expression of pak 2, promote the TGF-β signaling pathway, and ultimately inhibit OA by directly targeting PAK2 3′UTR. In summary, various miRNAs are involved in regulating OA progression by handling a variety of intracellular signaling pathways. In addition, increasing evidence also emphasizes that changes in the expression of many miRNAs can also directly regulate the development of OA. The specific information of these miRNAs is listed in Table 1.

**TABLE 1:** Functional characterization of the miRNAs in OA.

miRNA	Expression	Target gene(s)	Tissue/cell source	Region	Model	Functions	Reference
miR-103	Up	SPHK1	Cartilage tissue	knee joint, hip joint	OA rat model	Apoptosis	Li et al. (2019a)
	Up	Sox6	Cartilage tissue	knee joint	OA cell model	Apoptosis	Chen and Wu, (2019)
miR-34a	Up	TGIF2	Synovial fluid	knee joint	OA cell model	Apoptosis	Luo et al. (2019a)
	Up	DLL1	Cartilage tissue	knee joint, hip joint	OA rat model	Apoptosis	Zhang et al. (2018d)
	Up	*SIRT1/p53*	Cartilage tissue	knee joint	OA rat model	Apoptosis	Yan et al. (2016)
	Up	Cyr61	Cartilage tissue	knee joint	OA cell model	Apoptosis	Yang et al. (2018a)
	Up	——	Cartilage tissue	knee joint	OA rat model	Apoptosis	Tao et al. (2020)
	Up	——	Cartilage tissue	knee joint	OA rat model	Apoptosis	Abouheif et al. (2010)
miR-486-5p	Up	SMAD2	Cartilage tissue	knee joint	OA cell model	Apoptosis	Shi et al. (2018)
miR-375	Up	*JAK2*	Cartilage tissue	knee joint	OA mouse model	Apoptosis	Zou et al. (2019)
	Up	ATG2B	Cartilage tissue	knee joint	OA mouse model	Autophagy	Li et al. (2020c)
miR-29b	Up	PTHLH	Cartilage tissue	knee joint	OA mouse model	Apoptosis	Dou et al. (2020)
	Up	*Wnt5a*	——	——	OA mouse model	cartilage degradation	Sun et al. (2020)
	Up	COL2A1, COL1A2	Cartilage tissue	knee joint, hip joint	OA mouse model	Apoptosis	Moulin et al. (2017)
	Up	COL1A1, COL3A1	Cartilage tissue	knee joint, hip joint	OA cell model	Apoptosis	Mayer et al. (2017)
miR-29b-3p	Up	PGRN	Cartilage tissue	knee joint	OA rat model	Apoptosis	Chen et al. (2017)
miR-124A	Up	QKI, MAP 1B	Cartilage tissue	knee joint	OA rat model	cartilage degradation	Jiang et al. (2020b)
miR-455-3p	Up	PAK2	Cartilage tissue	knee joint	OA mouse model	cartilage degradation	Hu et al. (2019b)
	Up	COL2A1	Cartilage tissue	——	OA cell model	Apoptosis, Inflammation	Cheng et al. (2020)
	Up	PTEN	Bone marrow, Cartilage tissue	——	OA mouse model	Apoptosis, Inflammation	Wen et al. (2020)
miR-30b	Up	ERG	Cartilage tissue	knee joint	OA cell model	cartilage degradation	Li et al. (2015)
miR-181	Up	PTEN	Cartilage tissue	knee joint	OA cell model	Apoptosis	Wu et al. (2017b)
miR-324-5p	Up	Gpc1	Cartilage tissue	——	OA cell model	——	Woods et al. (2019)
miR-146a	Up	TRAF6	Cartilage tissue	knee joint, hip joint	OA cell model	Apoptosis	Zhong et al. (2017)
	Up	Camk2d, Ppp3r2	Cartilage tissue	knee joint	OA mouse model	cartilage degradation	Zhang et al. (2017)
	Up	Smad4	Cartilage tissue	knee joint	OA rat model	Apoptosis	Li et al. (2012)
	Up	CXCR4	Cartilage tissue	——	OA mouse model	nflammation	Sun et al. (2017)
miR-146a-5p	Up	TRAF6	Cartilage tissue	hip joint	OA cell model	Apoptosis	Shao et al. (2020)
	Up	TXNIP	SW1353 and C28/I2 cells	——	——	Apoptosis, Inflammation	Zhao and Gu, (2020)
	Up	——	Cartilage tissue, Blood	——	OA cell model	cartilage degradation, Inflammation	Skrzypa et al. (2019)
miR-146b	Up	A2M	Cartilage tissue	knee joint	OA mouse model	Apoptosis, cartilage degradation	Liu et al. (2019d)
	Up	——	Bone marrow, Cartilage tissue	——	OA cell model	Apoptosis	Budd et al. (2017)
miR-1236	Up	rs4246215	Cartilage tissue	knee joint	OA cell model	Apoptosis	Wang et al. (2020b)
miR-10a-5p	Up	HOXA3	Cartilage tissue, Blood	——	OA mouse model	Apoptosis, cartilage degradation	Li et al. (2020b)
	Up	HOXA1	Cartilage tissue	hip joint	OA mouse model	Apoptosis	Ma et al. (2019b)
miR-27b-3p	Up	KDM4B	Cartilage tissue	knee joint	OA rat model	Inflammation	Zhang et al. (2020c)
miR-483-5p	Up	Matn3, Timp2	Cartilage tissue	knee joint	OA mouse model	cartilage degradation	Wang et al. (2017b)
miR-340-5p	Up	FMOD	Cartilage tissue	knee joint	OA mouse model	Apoptosis	Zhang et al. (2018c)
miR-195	Up	PTHrP	Cartilage tissue	knee joint	OA rat model	Apoptosis	Cao et al. (2019b)
miR-195-5p	Up	REGγ	Cartilage tissue	——	OA mouse model	Apoptosis	Shu et al. (2019)
miR-23b-3p	Up	COL11A2	Cartilage tissue	knee joint	OA mouse model	inflammation	Yang et al. (2019b)
miR-448	Up	matrilin-3	Cartilage tissue	knee joint	OA cell model	Apoptosis, cartilage degradation	Yang et al. (2018b)
miR-203	Up	ERα	Blood, Cartilage tissue	——	OA rat model	cartilage degradation	Tian et al. (2019)
	Up	MCL-1	Cartilage tissue	——	OA cell model	Apoptosis, cartilage degradation, Inflammation	Zhao et al. (2017)
miR-203a	Up	*Smad3*	Cartilage tissue	knee joint	OA cell model	cartilage degradation, Inflammation	An et al. (2020)
miR-21	Up	GDF-5	Cartilage tissue	——	OA cell model	Apoptosis	Zhang et al. (2014)
miR-21-5p	Up	FGF18	Cartilage tissue	knee joint	OA mouse model	Apoptosis, cartilage degradation	Wang et al. (2019e)
miR-218-5p	Up	PIK3C2A	Cartilage tissue	knee joint	OA mouse model	cartilage degradation, Apoptosis	Lu et al. (2017)
miR-449a	Up	GDF5	Cartilage tissue	——	OA cell model	cartilage degradation	Wu et al. (2018a)
miR-125b-5p	Up	SYVN1	Synovial fluid	——	OA cell model	Apoptosis	Ge et al. (2017)
miR-384-5p	Up	SOX9	Cartilage tissue	knee joint	OA mouse model	Apoptosis	Zhang et al. (2020i)
miR-23a-3p	Up	SMAD3	Cartilage tissue	——	OA cell model	cartilage degradation	Kang et al. (2016a)
miR-139	Up	MCPIP1	Cartilage tissue	——	OA cell model	Apoptosis	Makki and Haqqi, (2015)
miR-206	Up	——	Cartilage tissue	knee joint	OA cell model	Apoptosis	Ni et al. (2018)
miR-382-3p	Up	CX43	Cartilage tissue	knee joint	OA cell model	Inflammation	Lei et al. (2019)
miR-101	Up	Sox9	Synovial fluid	knee joint	OA rat model	cartilage degradation	Dai et al. (2015)
miR-30a	Up	Sox9	Cartilage tissue	knee joint	OA cell model	cartilage degradation, Inflammation	Chang et al. (2016)
	Up	DLL4	bone marrow	——	OA rat model	Cell differentiation	Tian et al. (2016)
miR-216b	Up	Smad3	Cartilage tissue	knee joint	OA cell model	cartilage degradation	He et al. (2017)
miR-128a	Up	Atg12	Cartilage tissue	knee joint	OA rat model	Autophagy	Lian et al. (2018)
miR-20a	Up	IkBβ	Cartilage tissue, blood	——	OA rat model	Inflammation	Zhao and Gong, (2019)
miR-136	Up	Mcl-1	Cartilage tissue	——	OA cell model	Apoptosis, cartilage degradation, Inflammation	Wang and Kong, (2018)
miR-130b	Up	SOX9	Bone marrow, Cartilage tissue	——	OA rat model	Cell differentiation	Zhang et al. (2021c)
miR-132-3p	Up	ADAMTS-5	Bone marrow, Cartilage tissue	——	OA rat model	Cell differentiation	Zhou et al. (2018c)
miR-1246	Up	HNF4γ	Cartilage tissue	——	OA mouse model	Inflammation	Wu et al. (2017a)
miR-9	Up	——	Cartilage tissue	——	OA mouse model	Apoptosis, cartilage degradation, Inflammation	Zhang et al. (2019e)
miR-222	Up	HDAC-4	Cartilage tissue	knee joint	OA mouse model	Apoptosis	Song et al. (2015)
miR-155	Up	PIK3R1	Cartilage tissue	knee joint	OA cell model	Apoptosis	Fan et al. (2020)
miR-33a	Up	Smad7	Cartilage tissue	knee joint	OA cell model	Cell differentiation	Kostopoulou et al. (2015)
miR-93	Down	TLR4	Cartilage tissue, Synovial fluid	knee joint	OA mouse model	Apoptosis, inflammation	Ding et al. (2019)
miR-93-5p	Down	TCF4	Cartilage tissue	knee joint	OA rat model	Apoptosis	Xue et al. (2019)
miR-92a-3p	Down	WNT5A	Bone marrow, Cartilage tissue	——	OA mouse model	cartilage degradation	Mao et al. (2018b)
miR-92a-3p	Down	HDAC2	Bone marrow, Cartilage tissue	——	OA cell model	cartilage degradation	Mao et al. (2017b)
miR-92a-3p	Down	ADAMTS-4, ADAMTS-5	Cartilage tissue	knee joint	OA cell model	cartilage degradation, Inflammation	Mao et al. (2017a)
miR-107	Down	TRAF3	Cartilage tissue	knee joint	OA rat model	Autophagy and apoptosis	Zhao et al. (2019b)
miR-101a-3p	Down	UBE2D1, FZD4	Cartilage tissue	——	OA rat model	Apoptosis	Mao et al. (2021a)
miR-671	Down	——	Cartilage tissue	knee joint	OA mouse model	Apoptosis	Zhang et al. (2019a)
miR-671-3p	Down	TRAF3	Cartilage tissue	knee joint	OA cell model	cartilage degradation, Inflammation, Apoptosis	Liu et al. (2019e)
miR-140	Down	——	Synovial fluid, Cartilage tissue	knee joint	OA cell model	cartilage degradation	Si et al. (2016)
	Down	RALA	Cartilage tissue	knee joint	OA cell model	Cell differentiation	Karlsen et al. (2014)
	Down	IGFBP-5	Cartilage tissue	knee joint	OA cell model	cartilage degradation	Tardif et al. (2009)
	Down	IGFBP5	Cartilage tissue	knee joint	OA cell model	inflammation	Karlsen et al. (2016)
	Down	ADAMTS5	Cartilage tissue	——	OA mouse model	cartilage degradation	Miyaki et al. (2010)
	Down	MMP-13	Cartilage tissue	——	OA cell model	cartilage degradation	(Liang et al., 2012; Liang et al., 2016)
	Down	SMAD1	Cartilage tissue	——	OA cell model	Apoptosis	Li et al. (2018a)
	Down	NFAT3, SMAD3	Cartilage tissue	knee joint	OA cell model	inflammation	Tardif et al. (2013)
miR-140-3p	Down	CXCR4	Cartilage tissue	knee joint	OA cell model	Apoptosis	Ren et al. (2020)
miR-140-5p	Down	SMAD3	——	——	OA mouse model	inflammation	Li et al. (2019d)
	Down	HMGB1	Cartilage tissue	knee joint	OA cell model	inflammation	Wang et al. (2020d)
	Down	FUT1	Cartilage tissue	knee joint	OA cell model	Apoptosis	Wang et al. (2018b)
miR-33b-3p	Down	DNMT3A	Cartilage tissue	knee joint	OA cell model	Apoptosis	Ma et al. (2019a)
miR-766-3p	Down	AIFM1	Cartilage tissue	——	OA cell model	cartilage degradation	Li et al. (2020g)
miR-26a	Down	——	Cartilage tissue	knee joint	OA rat model	inflammation	Zhao et al. (2019c)
miR-26a/miR-26b	Down	FUT4	Cartilage tissue	knee joint	OA rat model	Apoptosis	Hu et al. (2018a)
miR-26a-5p	Down	PTGS2	Bone marrow, Synovial fluid	——	OA rat model	Apoptosis, Inflammation	Jin et al. (2020)
miR-377-3p	Down	ITGA6	Cartilage tissue	knee joint	OA cell model	Apoptosis	Tu et al. (2020)
miR-410-3p	Down	HMGB1	Synovial fluid, Cartilage tissue	knee joint	OA mouse model	Apoptosis, Inflammation	Pan et al. (2020)
miR-142-3p	Down	HMGB1	Cartilage tissue	knee joint	OA mouse model	Apoptosis, Inflammation	Wang et al. (2016c)
miR-210	Down	HIF-3α	Cartilage tissue	knee joint	OA cell model	Apoptosis, cartilage degradation	Li et al. (2016)
	Down	DR6	Cartilage tissue	knee joint	OA rat model	Apoptosis, Inflammation	Zhang et al. (2015)
miR-122	Down	SIRT1	Cartilage tissue	knee joint	OA cell model	cartilage degradation	Bai et al. (2020a)
miR-337-3p	Down	PTEN	Cartilage tissue	knee joint	OA cell model	Apoptosis	Huang et al. (2017)
miR-129-3p	Down	CPEB1	Cartilage tissue	knee joint	OA rat model	Apoptosis	Chen et al. (2020d)
miR-675-3p	Down	GNG5	Cartilage tissue	knee joint	OA cell model	Apoptosis, cartilage degradation	Shen et al. (2020b)
miR-132	Down	PTEN	Cartilage tissue, Blood	——	OA rat model	Apoptosis	Zhang et al. (2021d)
miR-137	Down	TCF4	Cartilage tissue	knee joint	OA rat model	Apoptosis, inflammation	Wang et al. (2020a)
miR-320c	Down	*β*-catenin	Cartilage tissue	knee joint	OA mouse model	Apoptosis	Hu et al. (2019a)
miR-29a	Down	Bax	Cartilage tissue	——	OA cell model	Apoptosis	Miao et al. (2019)
	Down	VEGF	Synovial fluid	knee joint	OA cell model	cartilage degradation, Inflammation	Ko et al. (2017)
miR-193b-3p	Down	MMP-19	Cartilage tissue	knee joint	OA cell model	Inflammation	Chang et al. (2018)
miR-193b-3p	Down	HDAC3	Cartilage tissue	knee joint	OA mouse model	cartilage degradation	Meng et al. (2018)
miR-193b-5p	Down	HDAC7	Cartilage tissue	knee joint, hip joint	OA cell model	Inflammation	Zhang et al. (2019b)
miR-136-5p	Down	ELF3	Bone marrow, Cartilage tissue	——	OA mouse model	Apoptosis, cartilage degradation	Chen et al. (2020e)
miR-374a-3p	Down	WNT5B	——	——	OA cell model	Apoptosis	Shi and Ren, (2020)
miR-19b-3p	Down	GRK6	Cartilage tissue	knee joint, hip joint	OA cell model	cartilage degradation, Inflammation	Duan et al. (2019a)
miR-221-3p	Down	SDF1/CXCR4	Cartilage tissue	knee joint	OA cell model	cartilage degradation	Zheng et al. (2017)
miR-502-5p	Down	TRAF2	Cartilage tissue	knee joint, hip joint	OA cell model	cartilage degradation, Inflammation	Zhang et al. (2016b)
miR-31	Down	CXCL12	Cartilage tissue	——	OA cell model	Apoptosis	Dai et al. (2019)
miR-488	Down	ZIP-8	Cartilage tissue	knee joint	OA mouse model	cartilage degradation	Song et al. (2013)
miR-125b	Down	ADAMTS-4	Cartilage tissue	knee joint	OA cell model	——	Matsukawa et al. (2013)
miR-181c	Down	NEAT1	Synovial fluid	——	OA cell model	Apoptosis, Inflammation	Wang et al. (2017d)
miR-615-3p	Down	——	bone marrow	——	OA rat model	Inflammation	Zhou et al. (2018a)
miR-211-5p	Down	Fibulin-4	Cartilage tissue	——	OA rat model	cartilage degradation, Inflammation	Liu and Luo, (2019)
miR-19a	Down	SOX9	Cartilage tissue	knee joint	OA cell model	Apoptosis	Yu and Wang, (2018)
miR-503-5p	Down	SGK1	Cartilage tissue	knee joint	OA rat model	Apoptosis, Inflammation	Wang et al. (2021b)
miR-33	Down	CCL2	Cartilage tissue	——	OA mouse model	Inflammation	Wei et al. (2016)
miR-27	Down	Leptin	Cartilage tissue	——	OA rat model	Inflammation	Zhou et al. (2017)
miR-186	Down	SPP1	Cartilage tissue	——	OA mouse model	Apoptosis	Lin et al. (2019)
miR-149	Down	TAK1	Cartilage tissue	——	OA cell model	Inflammation	Chen et al. (2018a)
miR-204-5p	Down	Runx2	Cartilage tissue	knee joint	OA rat model	Apoptosis	Cao et al. (2018a)
miR-128-3p	Down	WISP1	Cartilage tissue	knee joint	OA cell model	Apoptosis, cartilage degradation, Inflammation	Chen and Li, (2020)
miR-320	Down	*MMP-13*	Cartilage tissue	——	OA mouse model	Inflammation	Meng et al. (2016)
miR-558	Down	COX-2	Cartilage tissue	knee joint	OA cell model	Inflammation	Park et al. (2013)
miR-634	Down	PIK3R1	Cartilage tissue	——	OA cell model	cartilage degradation	Cui et al. (2016)
miR-24	Down	C-myc	Cartilage tissue	knee joint	OA rat model	Apoptosis	Wu et al. (2018b)
miR-365	Down	HIF-2α	Cartilage tissue	knee joint	OA cell model	Apoptosis	Hwang et al. (2017)
miR-126-3p	Down	——	Synovial fluid	knee joint	OA rat model	cartilage degradation, Inflammation	Zhou et al. (2021d)
miR-520c-3p	Down	GAS2	Cartilage tissue	hip joint	OA cell model	Apoptosis, cartilage degradation	Peng et al. (2021)
miR-1207-5p	Down	CX3CR1	Cartilage tissue	——	OA cell model	Apoptosis, cartilage degradation	Liu et al. (2020b)
miR-152	Down	TCF-4	Cartilage tissue	knee joint, hip joint	OA rat model	Apoptosis	Wan et al. (2020)
miR-296-5p	Down	TGF-β	Cartilage tissue	knee joint	OA cell model	Apoptosis, cartilage degradation	Cao et al. (2020)
miR-373	Down	P2X7R	Cartilage tissue, Blood	——	OA cell model	cartilage degradation, Inflammation	Zhang et al. (2018e)
miR-25-3p	Down	IGFBP7	Cartilage tissue	——	OA rat model	Apoptosis	He and Deng, (2021)
miR-95-5p	Down	HDAC2, HDAC8	Bone marrow, Cartilage tissue	——	OA cell model	cartilage degradation	Mao et al. (2018a)
miR-181a	Down	GPD1L	Cartilage tissue	knee joint	OA cell model	Apoptosis	Zhai et al. (2017)
miR-411	Down	HIF-1α	Cartilage tissue	——	OA cell model	autophagy	Yang et al. (2020b)
miR-98	Down	Bcl-2	Cartilage tissue	——	OA mouse model	Apoptosis	Wang et al. (2017c)
	Down	Bcl-2	Cartilage tissue	——	OA rat model	cartilage degradation、Apoptosis	Wang et al. (2016b)
	Down	——	Cartilage tissue	knee joint	OA rat model	Apoptosis	Wang et al. (2016a)
miR-125b-5p	Down	TRAF6	Cartilage tissue	knee joint, hip joint	OA cell model	Inflammation	Rasheed et al. (2019)
miR-27a	Down	TLR4	Cartilage tissue	knee joint, hip joint	OA rat model	cartilage degradation、Inflammation	Qiu et al. (2019)
	Down	NF-κB	Cartilage tissue	knee joint	OA rabbit model	Apoptosis, Inflammation	Zhang et al. (2019c)
	Down	PLK2	Cartilage tissue	knee joint	OA rat model	Apoptosis	Liu et al. (2019c)
miR-15a-5p	Down	PTHrP	Cartilage tissue	knee joint	OA cell model	Apoptosis	Duan et al. (2019b)
miR-9-5p	Down	Tnc	Cartilage tissue	knee joint, hip joint	OA mouse model	Apoptosis	Chen et al. (2019a)
miR-145	Down	BNIP3	Cartilage tissue	knee joint	OA mouse model	Apoptosis	Wang et al. (2020c)
miR-145	Down	MKK4	Cartilage tissue	——	OA rat model	cartilage degradation	Hu et al. (2017)
miR-145	Down	TNFRSF11B	Cartilage tissue	knee joint	OA cell model	Apoptosis	Wang et al. (2017a)

Abbreviations: SPHK1, sphingosine kinase-1; SOX9, SRY-Box 9; DLL1, delta-like protein 1; SIRT1, silent information regulator 1; SMAD2, SMAD, family member 2; PTHLH, parathyroid hormone-like hormone; Wnt5a, wnt family member 5A; PGRN, progranulin; MAP, 1B, microtubule associated protein 1B; Gpc1, glypican 1; Ppp3r2, calcineurin B, type II, protein phosphatase 3; TXNIP, thioredoxin-interacting protein; A2M, alpha-2-macroglobulin; KDM4B, lysine demethylase 4B; Matn3, cartilage matrix protein matrilin 3; Timp2, tissue inhibitor of metalloproteinase 2; PTHrP, parathyroid hormone-related protein; MCL-1, myeloid cell leukemia-1; GDF-5, growth differentiation factor 5; FGF18, fibroblast growth factor 18; GDF5, growth differentiation factor 5; SYVN1, synoviolin 1; CX43, connexin 43; TLR4, toll-like receptor 4; TCF4, transcription factor 4; HDAC2, histone deacetylase 2; ADAMTS-4, aggrecanase-1; TRAF3, TNF, receptorassociated factor 3; UBE2D1, ubiquitin-conjugating enzyme 2D1; FZD4, frizzled class receptor 4; MMP-13, matrix metalloproteinase-13; FUT1, fucosyltransferase 1; DNMT3A, DNA, methyltransferase 3A; DR6, death receptor 6; GNG5, G-protein subunit g 5; HDAC7, histone deacetylase 7; HDAC3, histone deacetylase 3; ELF3, E74-like factor 3; TRAF2, TNF, receptorassociated factor 2; SPP1, phosphoprotein 1; COX-2, cyclooxygenase-2; HIF-2α, hypoxia-inducible factor-2α; GAS2, Growth arrest-specific 2; P2X7R, P2X7 receptor; IGFBP7, insulin-like growth factor-binding protein 7; HIF-1α, hypoxia-inducible factor 1 alpha; Bcl-2, B-cell lymphoma 2; PLK2, polo-like kinase 2.

## lncRNAs and OA

**FIGURE 2:**
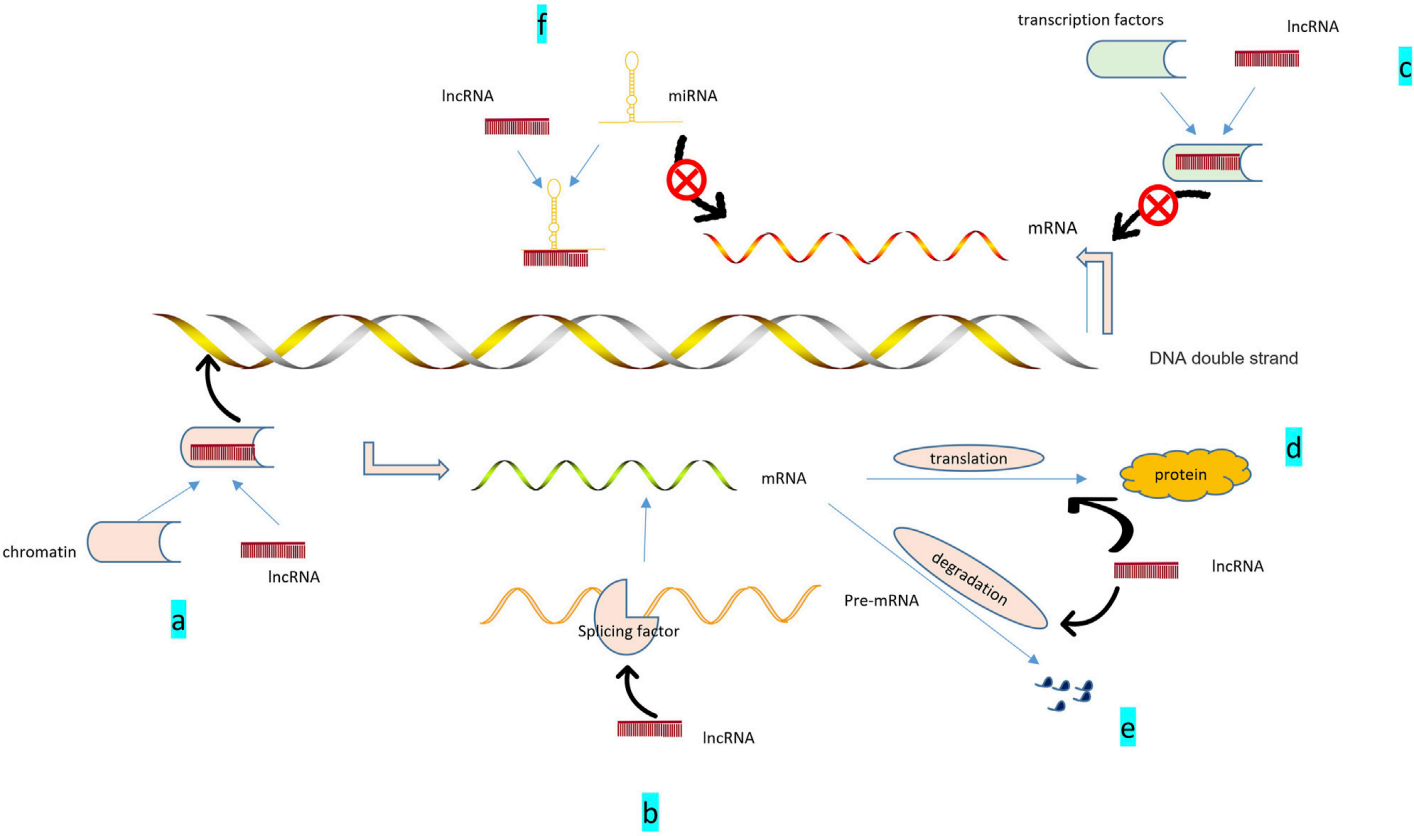
Role of lncRNAs: 1. Epigenetic regulation: **(A)**. lncRNA recruits chromatin remodeling and modification complexes to specific sites; regulates DNA or RNA methylation status, chromosome structure; and promotes the expression of related genes.2. Transcriptional regulation: **(B)**. lncRNA can help generate mature mRNA by promoting the binding of pre-mRNA to alternative splicing factors; **(C)**. ncRNA binding with transcription factors can inhibit the activity of target genes and inhibit their gene expression. 3. Post-transcriptional regulation: **(D)**. Participation in mRNA translation; **(E)**. Involvement in mRNA degradation. 4. Regulation of miRNA: **(F)**. ncRNA can act as sponges of miR-compete for miR and alleviate the inhibition of target genes.

lncRNAs are ncRNAs with a length of more than 200 nucleotides that have little or no protein-coding potential, and account for more than 80% of total lncRNAs(Ponting et al., 2009). At first, lncRNA was considered the “noise” of genome transcription, with no biological function, and its mechanism of action was only *in situ* regulation, through recruitment and formation of chromatin modification complexes [such as IGF2RRNA antisense (AIR), XIST] to silence the transcription of neighboring genes. As more detection techniques were applied to RNA studies, such as microarray, RNA sequencing (RNA-seq), Northern blot, and real-time quantitative reverse transcription-polymerase chain reaction (qRT-PCR) (Zhu et al., 2013), more biological functions of lncRNAs gradually being discovered. Recent studies have discovered several mechanisms of action of lncRNA, which can interact with proteins, DNA, and RNA to regulate many biological processes (Zhu et al., 2013). For example, lncRNA MALAT1 acts on miR-150-5P and AKT3 to regulate cell proliferation and apoptosis (Zhang et al., 2019g), thus participating in the growth and development of the body and the pathological process of diseases (Kopp and Mendell, 2018) (Figure 2).

lncRNA is closely related to cell growth, differentiation, and senescence. In addition, lncRNA has a special relationship with some human diseases, such as cardiovascular diseases (Huang, 2018), nervous system diseases (Zhang et al., 2019f), and immune-mediated diseases (Zhou et al., 2018b). In the recently updated database of lncRNA-related diseases, more than 200,000 lncRNAs have been recorded in their association with diseases (Bao et al., 2019).

lncRNA can regulate chondrocyte proliferation and apoptosis, inflammatory response, and extracellular matrix degradation, and promote the repair and stability of articular cartilage. Recent studies have shown an essential relationship between some changes or disorders of lncRNAs and the occurrence and development of OA. There are many studies to detect the expression of lncRNA in OA patients. Yang et al. (2021a) examined the lncRNA profiles of patients with OA and healthy individuals by RNA sequencing. They found that 25 lncRNAs are differentially expressed in patients with OA compared with the control group. Through microarray analysis, Xing et al. (2014) detected the expression of lncRNA in KOA cartilage and normal cartilage and further verified it by real-time polymerase chain reaction (RT-PCR). They found that the expression of 121 lncRNAs in KOA is different from normal cartilage: 73 up-regulated lncRNAs and 48 down-regulated lncRNAs. Among the up-regulated lncRNAs, HOTAIR is the most up-regulated. Pearson et al. (2016) separated OA chondrocytes through collagenase digestion and analyzed lncRNA expression through RNA sequencing (RNAseq) and qPCR. Finally, 983 lncRNAs were identified in OA chondrocytes. A total of 125 differentially expressed lncRNAs were identified after interleukin-1B (IL-1B) stimulation. Through microarray and qPCR analysis, Liu et al. (2014b) compared the expression of lncRNA in OA cartilage and normal cartilage, and found 152 differentially expressed lncRNAs in OA cartilage. Compared with normal cartilage, 82 increased lncRNAs and 70 decreased lncRNAs were in OA cartilage. Using mRNA and lncRNA microarray analysis, Zhang et al. (2020a) found that 990 lncRNAs were different in OA chondrocytes compared with the control group: 666 up-regulated, 324 down-regulated. In addition, 546 mRNAs had a different expression: 419 up-regulated, 127 down-regulated. Six lncRNAs (ENST00000606283.1, ENST00000436872.1, ENST00000488584.1, ENST00000603682.1, XR-245446.2, and ENST00000605586.1) were tested by qPCR. The results were consistent with the test results. In summary, through the detection of lncRNA expression levels in the chondrocytes of OA patients and healthy individuals, we can finally find that there are differences in the expression of a variety of lncRNAs. In addition to the lncRNAs of appeal, several lncRNAs are closely related to the progress of OA, as shown in Table 2.

**TABLE 2:** Functional characterization of the lncRNAs in OA.

lncRNA	Expression	Target genes	Related genes	Tissue/cell source	Region	Model	Functions	Reference
ANRIL	Up	miRNA-122-5p	DUSP4	Cartilage tissue, synoviocytes	knee joint	OA cell model	Cell proliferation and apoptosis	Li et al. (2019e)
CASC2	Up	——	IL-17	Blood, Synovial fluid, chondrocyte	——	OA cell model	Cell proliferation and apoptosis	Huang et al. (2019d)
CIR	Up	——	——	Cartilage tissue	hip joint	OA rat model	Cell autophagy	Wang et al. (2018a)
	Up	miR-130a	Bim	Cartilage tissue	knee joint	OA cell model	Cell proliferation and apoptosis	Lu et al. (2018)
	Up	miR-27b	——	Cartilage tissue	knee joint	OA cell model	Degradation of extracellular matrix	Li et al. (2017b)
HOTAIR	Up	——	MMP	Cartilage tissue, Synovial fluid	temporomandibular	OA rabbit model	Cell proliferation and apoptosis	Zhang et al. (2016a)
	Up	——	WIF-1	SW1353 cells	knee joint	OA cell model	Degradation of extracellular matrix	Yang et al. (2020c)
	Up	——	——	Synovial fluid, Cartilage tissue	knee joint	OA cell model	Cell proliferation and apoptosis	Liang et al. (2021)
	Up	——	——	Synovial tissue	knee joint	0A rat model	Cell proliferation and apoptosis, inflammation	Mao et al. (2019b)
	Up	miR-20b	PTEN	Cartilage tissue	knee joint	OA mouse model	Cell proliferation and apoptosis, Degradation of extracellular matrix	Chen et al. (2020f)
	Up	miR-130a-3p	——	Cartilage tissue	——	OA human model	Cell proliferation and apoptosis, Cell autophagy	He and Jiang, (2020)
	Up	miR-17-5p	FUT2	Cartilage tissue	knee joint	0A rat model	Cell proliferation and apoptosis, Degradation of extracellular matrix	Hu et al. (2018b)
LOC101928134	Up	——	IFNA1	Synovial fluid, Cartilage tissue	knee joint	0A rat model	Cell proliferation and apoptosis, Degradation of extracellular matrix	Yang et al. (2019a)
LINC00671	Up	——	Smurf2	Cartilage tissue	knee joint	OA mouse model	Degradation of extracellular matrix	Chen and Xu, (2021)
TM1P3	Up	——	——	Cartilage tissue	knee joint	OA cell model	Degradation of extracellular matrix	Li et al. (2019g)
GAS5	Up	miR-144	mTOR	Cartilage tissue	knee joint	0A rat model	Cell proliferation and apoptosis	Ji et al. (2021)
	Up	miR-137	——	Blood, cartilage tissues	——	OA cell model	Cell proliferation and apoptosis	Gao et al. (2020)
	Up	miR-21	——	Cartilage tissue	knee joint	OA cell model	Cell proliferation and apoptosis	Song et al. (2014)
SAMD14-4	Up	——	COL1A1, COL1A2	Cartilage tissue	knee joint	OA cell model	inflammation	Zhang et al. (2019d)
KLF3-AS1	Up	miR-206	GIT1	Cartilage tissue	knee joint	OA mouse model	Cell proliferation and apoptosis	Liu et al. (2018)
CTBP1-AS2	Up	miR-130a	——	Cartilage tissue, Synovial fluid	knee join、hip join	OA cell model	Cell proliferation and apoptosis	Zhang et al. (2020d)
H19	Up	miR-140-5p	——	Cartilage tissue	knee joint	OA cell model	Degradation of extracellular matrix	Yang et al. (2020a)
	Up	miR-675	——	Cartilage tissue	knee joint	OA cell model	Degradation of extracellular matrix	Steck et al. (2012)
	Up	miR-106b-5p	TIMP2	Cartilage tissue, Synovial fluid	knee joint	OA cell model	Degradation of extracellular matrix	Tan et al. (2020)
	Up	miR-29a-3p	FOS	Astrocytes	——	OA rat model	inflammation	Yang et al. (2021b)
PART1	Up	miR-373-3p	SOX4	Cartilage tissue	——	OA cell model	Cell proliferation and apoptosis, Degradation of extracellular matrix	Zhu and Jiang, (2019)
LOXL1-AS1	Up	miR-423-5p	KDM5C	Cartilage tissue	knee join, hip join	OA cell model	Cell proliferation and apoptosis	Chen et al. (2020c)
MALAT1	Up	miR-145	ADAMTS5	Cartilage tissue	——	OA cell model	Degradation of extracellular matrix	Liu et al. (2019a)
	Up	miR-146a-PI3K	——	Cartilage tissue	——	OA rat model	Degradation of extracellular matrix	Li et al. (2020d)
TUG1	Up	miR-195	MMP-13	Cartilage tissue	knee joint	OA cell model	Degradation of extracellular matrix	Tang et al. (2018b)
	Up	miR-320c	MMP-13	Cartilage tissue	knee joint	OA cell model	Degradation of extracellular matrix, Cell proliferation and apoptosis	Han and Liu, (2021)
XIST	Up	miR-211	CXCR4	Cartilage tissue	knee joint	OA cell model	Cell proliferation and apoptosis	Li et al. (2018b)
	Up	miR-149-5p	DNMT3A	Cartilage tissue	knee joint	OA cell model	Degradation of extracellular matrix	Liu et al. (2020c)
	Up	miR-1277-5p	——	Cartilage tissue	knee join, hip join	OA rat model	Degradation of extracellular matrix	Wang et al. (2019d)
FOXD2-AS1	Up	miR-27a-3p	TLR4	Cartilage tissue	knee joint	OA cell model	Cell proliferation and apoptosis	Wang et al. (2019f)
	Up	miR-206	CCND1	Cartilage tissue	knee joint	OA cell model	Cell proliferation and apoptosis	Cao et al. (2018b)
NEAT1	Up	miR-543	PLA2G4A	Cartilage tissue	knee joint	OA cell model	Cell proliferation and apoptosis	Xiao et al. (2021)
	Up	miR-16-5p	——	ATDC5	knee joint	OA cell model	Cell proliferation and apoptosis	Li et al. (2020a)
	Up	miR-193a-3p	SOX5	Cartilage tissue	knee joint	OA cell model	Degradation of extracellular matrix	Liu et al. (2020a)
IGHCγ1	Up	miR-6891-3p	TLR4	PBMCs	——	OA cell model	inflammation	Zhang et al. (2020g)
LINC00511	Up	miR-150-5p	SP1	ATDC5	——	OA cell model	Cell proliferation and apoptosis	Zhang et al. (2020m)
PVT1	Up	miR-488-3p	——	Cartilage tissue	knee joint	OA cell model	Cell proliferation and apoptosis	Li et al. (2017c)
	Up	miR-27b-3p	TRAF3	Cartilage tissue	——	OA cell model	inflammation	Lu et al. (2020)
	Up	miR-26b	——	Cartilage tissue	knee joint	OA cell model	Degradation of extracellular matrix	Ding et al. (2020)
	Up	miR-149	——	Cartilage tissue	knee joint	OA cell model	inflammation	Zhao et al. (2018)
	Up	miR-211-3p	——	SW982 cells, Chondrocytes	——	OA rat model	Cell proliferation and apoptosis	Xu et al. (2020)
CASC19	Up	miR-152-3p	DDX6	Cartilage tissue	——	OA cell model	inflammation	Zhou et al. (2021a)
CHRF	Up	miR-146a	——	ATDC5	——	OA cell model	inflammation	Yu et al. (2019a)
HOTTIP	Up	miR-663a	——	Cartilage tissue	knee joint	OA cell model	Cell proliferation and apoptosis	He et al. (2021b)
	Up	miR-455-3p	CCL3	Chondrocytes, Bone marrow	knee join, hip join	OA cell model	Degradation of extracellular matrix	Mao et al. (2019a)
DANCR	Up	miR-216a-5p	JAK2, STAT3	Cartilage tissue	knee joint	OA cell model	Cell proliferation and apoptosis	Zhang et al. (2018b)
	Up	miR-1275	MMP-13	SFMSCs, Synovial fluid	——	OA cell model	Cell proliferation and apoptosis	Fang et al. (2019)
	Up	miR-577	——	Cartilage tissue	knee join, hip join	OA cell model	Cell proliferation and apoptosis	Fan et al. (2018)
TNFSF10	Up	miR-376-3p	FGFR1	Cartilage tissue	knee joint	OA cell model	Cell proliferation and apoptosis	Huang et al. (2019a)
ARFRP1	Up	miR-15a-5p	TLR4	Cartilage tissue	knee joint	OA cell model	inflammation	Zhang et al. (2020b)
LINC00461	Up	miR-30a-5p	——	Cartilage tissue	——	OA cell model	Cell proliferation and apoptosis	Zhang et al. (2020n)
BLACAT1	Up	miR-142-5p	——	BMSCs, Bone marrow	——	OA rat model	Cell proliferation and apoptosis	Ji et al. (2020)
MCM3AP-AS1	Up	miR-1423p	HMGB1	Synovial fluid, chondrocyte	knee join, hip join	OA cell model	Cell proliferation and apoptosis	Gao et al. (2019b)
MCM3AP-AS1	Down	miR-138-5p	SIRT1	Cartilage tissue	knee joint	OA cell model	inflammation	Shi et al. (2021a)
PCAT-1	Up	miR-27b-3p	——	Cartilage tissue	knee join, hip join	OA cell model	Cell proliferation and apoptosis	Zhou et al. (2021c)
PMS2L2	Up	miR-203	——	ATDC5	——	OA cell model	inflammation	Li et al. (2019f)
LINC01534	Up	miR-140-5p	——	Cartilage tissue	knee joint	OA cell model	inflammation	Wei et al. (2019)
MIR22HG	Up	miR-9-3p	ADAMTS5	Cartilage tissue	knee joint	OA cell model	Degradation of extracellular matrix	Long et al. (2021)
PCGEM1	Up	miR-770	——	Synovial fluid	——	OA mouse model	Cell proliferation and apoptosis	Kang et al. (2016b)
DILC	Down	——	IL-6	Blood, Synovial fluid	——	OA cell model	inflammation	Huang et al. (2019b)
PACER	Down	——	HOTAIR	Blood	——	OA cell model	Cell proliferation and apoptosis	Jiang et al. (2019)
MIR4435-2HG	Down	——	——	Blood, Synovial fluid	knee joint	OA cell model	Cell proliferation and apoptosis	Xiao et al. (2019b)
HAND2-AS1	Down	——	IL-6	Blood, Synovial fluid	knee joint	OA cell model	inflammation	Si et al. (2021)
ANCR	Down	——	TGF-β1	Blood	——	OA cell model	Cell proliferation and apoptosis	Li et al. (2019c)
ROR	Down	——	——	Cartilage tissue	knee joint	OA cell model	Cell proliferation and apoptosis	Yang et al. (2018c)
FAS-AS1	Down	——	——	Cartilage tissue	knee joint	OA cell model	Degradation of extracellular matrix	Zhu et al. (2018)
lncRNA-NR024118	Down	——	——	ATDC5	——	OA mouse model	inflammation	Mei et al. (2019)
FER1L4	Down	——	IL-6	Blood, Synovial fluid	——	OA cell model	inflammation	He et al. (2021a)
ZFAS1	Down	——	——	Cartilage tissue	knee joint	OA cell model	Cell proliferation and apoptosis	Ye et al. (2018)
MEG3	Down	——	VEGF	Cartilage tissue	knee joint	OA cell model	Degradation of extracellular matrix	Su et al. (2015)
	Down	——	TRIB2	Synovial fluid	knee joint	OA cell model	Cell proliferation and apoptosis	You et al. (2019)
	Down	miR-361-5p	FOXO1	Cartilage tissue	knee joint	OA cell model	Cell proliferation and apoptosis	Wang et al. (2019a)
	Down	miR-16	SMAD7	Cartilage tissue	——	OA rat model	Cell proliferation and apoptosis	Xu and Xu, (2017)
	Down	miR-93	TGFBR2	Cartilage tissue	knee joint	OA rat model	Degradation of extracellular matrix	Chen et al. (2019b)
MALAT-1	Down	——	——	Cartilage tissue	knee joint	OA rat model	Cell proliferation and apoptosis	Gao et al. (2019a)
SNHG7	Down	miR-34a-5p	SYVN1	Cartilage tissue	knee joint	OA cell model	Cell proliferation and apoptosis	Tian et al. (2020)
	Down	miR-214-5p	PPARGC1B	Cartilage tissue	knee joint	OA cell model	inflammation	Xu et al. (2021)
SNHG9	Down	miR-34a	——	Cartilage tissue, Synovial fluid	knee joint	OA cell model	Cell proliferation and apoptosis	Zhang et al. (2020e)
NKILA	Down	miR-145	SP1	Cartilage tissue	——	OA cell model	Cell proliferation and apoptosis	Xue et al. (2020)
SNHG5	Down	miR-10a-5p	H3F3B	Cartilage tissue	knee joint	OA cell model	Cell proliferation and apoptosis	Jiang et al. (2020a)
	Down	miR-26a	SOX2	Cartilage tissue	knee joint	OA cell model	Cell proliferation and apoptosis	Shen et al. (2018)
PART-1	Down	miR-590-3p	TGFBR2/Smad3	Cartilage tissue	knee join, hip join	OA cell model	Cell proliferation and apoptosis	Lu et al. (2019)
OIP5-AS1	Down	miR-29b-3p	PGRN	Cartilage tissue	knee joint	OA cell model	inflammation	Zhi et al. (2020)
	Down	miR-30a-5p	——	Cartilage tissue	——	OA cell model	Cell proliferation and apoptosis	Qin et al. (2021)
DNM3OS	Down	miR-126	CHON-001	Cartilage tissue	knee joint	OA cell model	Cell proliferation and apoptosis	
LINC00623	Down	miR-101	HRAS	Cartilage tissue	knee joint	OA cell model	Cell proliferation and apoptosis	Lü et al. (2020)
ATB	Down	miR-223	——	ATDC5	——	OA mouse model	inflammation	Ying et al. (2019)
HOTAIRM1-1	Down	miR-125b	BMPR2	Cartilage tissue	knee joint	OA cell model	Cell proliferation and apoptosis	Xiao et al. (2019c)
HULC	Down	miR-101	——	Cartilage tissue	knee joint	OA cell model	inflammation	Chu et al. (2019)
SNHG15	Down	miR-141-3p	BCL2L13	Cartilage tissue	knee joint	OA rat model	Cell proliferation and apoptosis	Zhang et al. (2020k)
LINC00662	Down	miR-15b-5p	GPR120	Cartilage tissue	knee joint	OA rat model	inflammation	Lu and Zhou, (2020)
LUADT1	Down	miR-34a	SIRT1	Synovial fluid, chondrocytes	knee join, hip join	OA cell model	Cell proliferation and apoptosis	Ni et al. (2020b)
UFC1	Down	miR-34a	——	Cartilage tissue	knee joint	OA cell model	Cell proliferation and apoptosis	Zhang et al. (2016c)

Abbreviations: PBMCs, peripheral blood mononuclear cells; MMP, metalloproteinases; WIF-1, Wnt inhibitory factor 1; FUT2, fucosyltransferase 2; KDM5C, lysine demethylase 5C; DNMT3A, DNA, methyltransferase 3A; SIRT1, silent information regulator-1; TRIB2, Tribbles homolog 2; TGFBR2, transforming growth factor β receptor type II; KLF4, Krüppel-like factor 4; PPARGC1B, PPARG, coactivator 1 beta; H3F3B, H3 histone family 3B; Smad3, SMAD, family member 3; PGRN=progranulin; DNM3OS, dynamin 3 opposite strand; HRAS, Harvey rat sarcoma viral oncogene homolog; BMPR2, bone morphogenetic protein receptor 2.

## circRNA and OA

**FIGURE 3:**
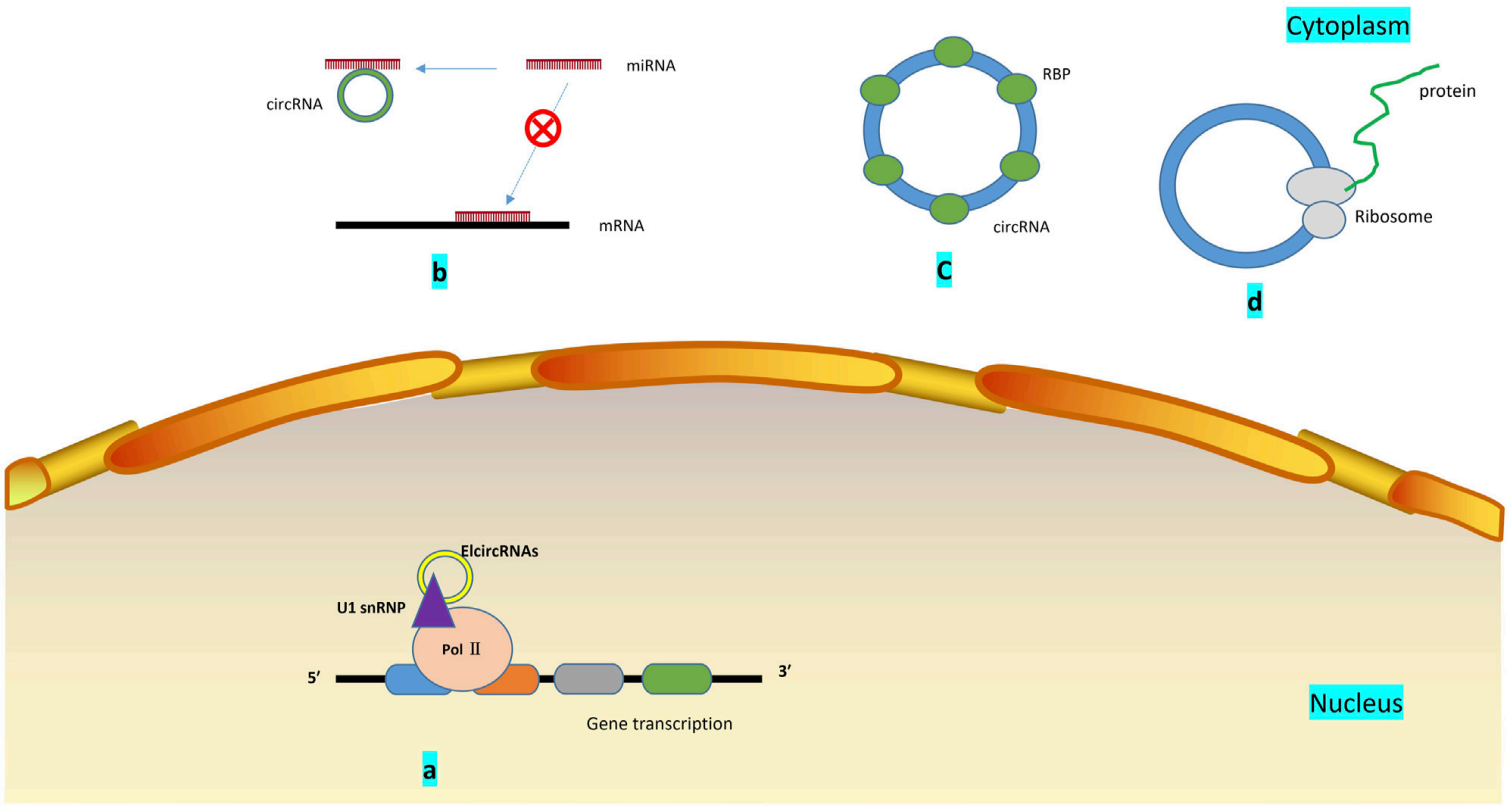
Biological functions of circRNA: **(A)**. Regulation of gene transcription: Elcircrna can interact with small nuclear ribonucleoproteins and bind to RNA polymerase II; **(B)**. miRNA sponge: circRNA contains miRNA binding sites, which can block miRNA binding to mRNA and promote or inhibit the expression of related genes by sponging miRNA; **(C)**. circRNAs bind to mRNA regulatory binding protein, which influences the stability of mRNA, and may change the splicing pattern of circRNA; **(D)**. By being translated by ribosomes and encoding polypeptides, several circRNAs can play a role in regulating and controlling human physiological processes.

The circRNA molecule is in a closed-loop structure and is not affected by RNA exonuclease. They are mainly in the cytoplasm or stored in exosomes. They are stable and not easily degradable, and widely exist in many eukaryotes. circRNAs are formed by reverse splicing through nonclassical splicing. One model believes that in the transcription of pre-RNA, due to the partial folding of RNA, the originally nonadjacent exons are pulled closer, and exon jumping occurs, resulting in the formation of circular RNA intermediates in the region to be crossed. Moreover, ring RNA molecules composed of exons are formed by lasso splicing. Another model suggests that the reverse complementary sequence located in the intron region leads to intron region pairing mediated reverse splicing, resulting in the formation of circular RNA molecules (Chen and Yang, 2015). To date, the biological functions of circRNAs that have been discovered mainly include interactions with miRNAs(Cao et al., 2019a), binding of regulatory proteins (Zang et al., 2020), transcription of regulatory genes (Zhang, 2020), and coding functions (Lei et al., 2020) (Figure 3). For example, circRNA.33186 increased MMP-13 expression by interacting with miR-127-5p to regulate cell proliferation and apoptosis (Zhou et al., 2019b).

Bipartite Network Projection allocates resources according to the known associations between different miRNAs and diseases, entirely using the similarity information of miRNA and diseases to predict various conditions accurately (Chen et al., 2018b). KATZ Measure is a graph-based calculation method, which converts the calculation of the similarity between lncRNA and diseases into the problem of similarity calculation between nodes in heterogeneous networks to predict the correlation between lncRNA and conditions. The integration of the two can recognize the association of circRNA with the disease (Chen, 2015). Through Bipartite Network Projection and KATZ Measure (Zhao et al., 2019a), many circRNAs related to diseases have been discovered, and circRNAs are involved in the diagnosis and treatment of atherosclerosis (Zhang et al., 2018a), cancer (Li et al., 2020f), cardiac hyperplasia (Li et al., 2020e), and other diseases. There are many experimental studies related to circRNA and diseases, and the main research types are cell experiments or animal experiments. Through these experiments, we have found multiple action mechanisms of circRNA on various conditions. For example, circRNA_100367 acts as a signaling molecule that regulates esophageal squamous cell carcinoma through the Wnt3 signaling pathway (Liu et al., 2019b); circRNA_0016624 regulates gene-based expression of interest in osteoporosis patients *via* sponge miR-98 (Yang et al., 2020d); circRNA_100395 mitigates the progression of breast cancer by directly targeting MAPK6 (Yu et al., 2020).

In addition, several circRNAs participate in the development of OA and the OA of the abnormal expression in various tissues. For example, Xiao et al. (2019a) used illumina sequencing platform to detect circRNA expression in patients with mild and severe KOA. In this paper, 197 differentially expressed circRNAs were identified. Among them, the up-regulation amplitude of Hg38_circ_0007474 is the largest, and the down-regulation amplitude of hg38_circ_0000118 is the largest. Further analysis of the three circRNAs selected from hsa_circ_0045714, hsa_circ_0005567, and hsa_circ_0002485 found that all three circRNAs can inhibit the function of the corresponding miRNA by serving as a sponge for miRNAs and indirectly promote its downstream process, thereby participating in the development of OA. Wang et al. (2019h) used microarray analysis to screen for circRNA expression in healthy and KOA articular cartilage. They found 1,380 circRNAs differentially expressed in the articular cartilage of knee joints of healthy individuals and patients with OA. Meanwhile, constructing a circRNA-miRNA network verified the ten most likely target genes related to circRNA. It was finally discovered that hsa_circ RNA_003231 might be involved in the occurrence and progression of OA. Zhou et al. (2018e) established OA models in interleukin-1β (IL1β)-treated mouse articular chondrocytes (MACs) to study the expression and function of circRNAs in OA using new sequencing methods and bioinformatic analysis. Compared with the control group, 255 circRNAs were differentially expressed in MACs treated with IL-1 β: 119 up-regulated, 136 down-regulated. Mmu-circRNA-30365 and Mmu-circRNA36866 were two substantially different circRNAs, and their specific expression changes in patients with OA and normal individuals were verified by QRT-PCR. Liu et al. (2016) analyzed circRNA expression between OA and normal cartilage samples by hierarchical clustering analysis and found that compared with normal cartilage, 71 circRNAs were differentially expressed (16 were increased, and 55 were decreased) in OA cartilage. In this study, we focused on the research of circRNA-CER. We found that this circRNA could compete with MMP13 for miR-136 and participate in the degradation of the extracellular matrix of chondrocytes. The above examples fully prove that the expression levels of circRNA in OA patients and healthy individuals are different, and these differentially expressed circRNA has a special relationship with the progression of OA.

Several studies have reported the functions and mechanisms of several circRNAs in OA, but relevant studies are few. Zhou et al. (2018d) established rat OA models, predicted the function of circRNA_ATP9b in rat knee chondrocytes through bioinformatic analysis, and finally found that circRNA_ATP9b regulated the degradation of extracellular matrix through sponge miR-138-5p, thereby controlling the progression of OA. Moreover, circRNA_ATP9b expression was increased, and miR-138-5p expression was down-regulated in IL-1β-induced chondrocytes. circRNA_ATP9b regulated the expression of related genes by targeting miR-138-5p. Li et al. (2017a) analyzed the dual-luciferase reporter genes and found that the transcriptional activity of miR-193b can be inhibited by overexpression of hsa_circ_0045714. Overexpression of hsa_circ_0045714 can also up-regulate the expression of insulin-like growth factor 1 receptor (IGF1R) because IGF1R is a crucial target gene of miR-193b. It is associated with cell proliferation and apoptosis. Further studies on the progression of circRNA in OA are presented in Table 3.

**TABLE 3:** Functional characterization of the circRNAs in OA.

CircRNA	Expression	Target genes	Related genes	Tissue/cell source	Region	Model	Functions	Reference
CircVCAN	Up	——	——	Cartilage tissue	——	OA cell model	Cell proliferation and apoptosis	Ma et al. (2020)
hsa_circ_0000448	Up	——	——	Synovial tissues	Temporomandibular joint	OA cell model	Degradation of extracellular matrix	Hu et al. (2019c)
hsa_circ_0037658	Up	——	——	Cartilage tissue	——	OA cell model	Cell autophagy	Sui et al. (2021)
hsa_circ_0032131	Up	——	——	Blood	——	OA cell model	Cell proliferation and apoptosis	Wang et al. (2019g)
	Up	miR-502-5p	PRDX3	Cartilage tissue	——	OA rat model	Cell proliferation and apoptosis	Xu and Ma, (2021)
CircRNA.33186	Up	miR-127-5p	——	Cartilage tissue	knee joint	OA mouse model	Cell proliferation and apoptosis	Zhou et al. (2019b)
CircRNA_0092516	Up	miR-337-3p	PTEN	Cartilage tissue	knee joint	OA mouse model	Cell proliferation and apoptosis	Huang et al. (2021)
CircGCN1L1	Up	miR-330-3p	TNF-α	Synovial fluid	Temporomandibular joint	OA rat model	Cell proliferation and apoptosis	Zhu et al. (2020)
CircRNA-UBE2G1	Up	miR-373	HIF-1a	Cartilage tissue	knee joint	OA cell model	Cell proliferation and apoptosis	Chen et al. (2020b)
CircRNA HIPK3	Up	miR-124	SOX8	Cartilage tissue	——	OA cell model	Cell proliferation and apoptosis	Wu et al. (2020)
CircTMBIM6	Up	miR-27a	MMP13	Cartilage tissue	knee joint	OA cell model	Degradation of extracellular matrix	Bai et al. (2020b)
CircPSM3	Up	miRNA-296-5p	——	Cartilage tissue	——	OA cell model	Cell proliferation and apoptosis	Ni et al. (2020a)
hsa_circ_0005105	Up	miR-26a	NAMPT	Cartilage tissue	——	OA cell model	Degradation of extracellular matrix	Wu et al. (2017c)
CircRNA-CDR1as	Up	miRNA-641	——	Cartilage tissue	knee joint	OA cell model	Degradation of extracellular matrix	Zhang et al. (2020j)
CircRNA Atp9b	Up	miR-138-5p	——	Cartilage tissue	knee joint	OA mouse model	Degradation of extracellular matrix	Zhou et al. (2018d)
Circ_0116061	Up	miR-200b-3p	SMURF2	Cartilage tissue	knee joint	OA cell model	Cell proliferation and apoptosis, inflammation	Zheng et al. (2021)
Circ-BRWD1	Up	miR-1277	TRAF6	Cartilage tissue	knee joint	OA cell model	Cell proliferation and apoptosis, Degradation of extracellular matrix, inflammation	Guo et al. (2021)
Circ-SPG11	Up	miR-337-3p	ADAMTS5	Cartilage tissue	knee joint	OA cell model	Cell proliferation and apoptosis, Degradation of extracellular matrix, inflammation	Liu et al. (2021b)
Circ_SLC39A8	Up	miR-591	IRAK3	Cartilage tissue	knee joint	OA cell model	Cell proliferation and apoptosis, Degradation of extracellular matrix, inflammation	Yu et al. (2021)
Circ-PRKCH	Up	miR-140-3p	ADAM10	Cartilage tissue	knee joint	OA cell model	Degradation of extracellular matrix	Zhao et al. (2021)
CircCDH13	Up	miR-296-3p	PTEN	Cartilage tissue	hip joint	OA mouse model	Cell proliferation and apoptosis, Degradation of extracellular matrix	Zhou et al. (2021e)
Circ-IQGAP1	Up	miR-671-5p	TCF4	Cartilage tissue	knee joint, hip joint	OA cell model	Cell proliferation and apoptosis, Degradation of extracellular matrix, inflammation	Xi et al. (2021)
Circ_0136,474	Up	miR-127-5p	MMP-13	Cartilage tissue	knee joint	OA cell model	Cell proliferation and apoptosis	Li et al. (2019i)
Circ_RUNX2	Up	——	RUNX2	Blood	——	OA cell model	Degradation of extracellular matrix	Wang et al. (2021a)
CircRSU1	Up	miR-93-5p	MAP3K8	Cartilage tissue	knee joint	OA mouse model	Degradation of extracellular matrix	Yang et al. (2021c)
CircRNA3503	Down	——	——	Synovial fluid	——	OA cell model	Degradation of extracellular matrix	Tao et al. (2021)
CircPDE4B	Down	——	RIC8A, MID1	Cartilage tissue	——	OA mouse model	Degradation of extracellular matrix	Shen et al. (2021)
CircSERPINE2	Down	miR-1271	——	Cartilage tissue	——	OA cell model	Degradation of extracellular matrix, Cell proliferation and apoptosis	Shen et al. (2019)
	Down	miR-495	TGFBR2	Cartilage tissue	knee joint	OA cell model	Cell proliferation and apoptosis	Zhang et al. (2020h)
CiRS-7	Down	miR-7	——	Cartilage tissue	——	OA rat model	Inflammation	Zhou et al. (2020a)
	Down	miR-7	——	Blood	——	OA cell model	Cell proliferation and apoptosis	Zhou et al. (2019a)
CircCDK14	Down	miR-125a-5p	Smad2	Cartilage tissue	knee joint	0A rabbit model	Cell proliferation and apoptosis	Shen et al. (2020a)
CircPDE4D	Down	miR-103a-3p	FGF18	Cartilage tissue	knee joint	OA mouse model	Degradation of extracellular matrix	Wu et al. (2021b)
CircRNA_0001236	Down	miR-3677-3p	Sox9	Bone marrow, Cartilage tissue	——	OA mouse model	Degradation of extracellular matrix	Mao et al. (2021b)
CircRNA-9119	Down	miRNA-26a	PTEN	Cartilage tissue	——	OA cell model	Cell proliferation and apoptosis	Chen et al. (2020a)
Hsa_circ_0005567	Down	miR-495	ATG14	Cartilage tissue	——	OA cell model	Cell autophagy and apoptosis	Zhang et al. (2020f)
CircRNA-CER	Up	MiR-136	MMP13	——	knee joint	OA cell model	Degradation of extracellular matrix	Liu et al. (2016)
CircHYBID	Down	hsa-miR-29b-3p	TGF-β1	Cartilage tissue	knee joint	OA cell model	Degradation of extracellular matrix	Liao et al. (2021)
CircADAMTS6	Down	miR-431-5p	——	Cartilage tissue	——	OA cell model	Cell proliferation and apoptosis	Fu et al. (2021)
Hsa_circ_0045714	Down	miR-193b	IGF1R	Cartilage tissue	knee joint	OA cell model	Cell proliferation and apoptosis	Li et al. (2017a)
Circ_0020093	Down	miR-23b	SPRY1	Cartilage tissue	——	OA cell model	Degradation of extracellular matrix	Feng et al. (2021)
CircANKRD36	Down	miR-599	Casz1	Cartilage tissue	——	OA cell model	Cell proliferation and apoptosis	Zhou et al. (2021b)
CircSLC7A2	Down	miR-4498	TIMP3	Cartilage tissue	——	OA mouse model	Degradation of extracellular matrix, Cell proliferation and apoptosis, inflammation	Ni et al. (2021)

Abbreviations: TNF-α, tumor necrosis factor-α; LEF1, lymphoid enhancer-binding factor 1; NAMPT, nicotinamide phosphoribosyltransferase; SMURF2, Smad ubiquitin regulatory factor 2; TRAF6, TNF, receptorassociated factor 6; ADAM10, a-disintegrin and metallopeptidase domain 10; PTEN, phosphatase and tensin homolog; MID1, midline 1; TGFBR2, transforming growth factor-β receptor 2; SPRY1, sprouty 1.

## Interactions Between lncRNAs, miRNAs and mRNAs in OA

**FIGURE 4:**
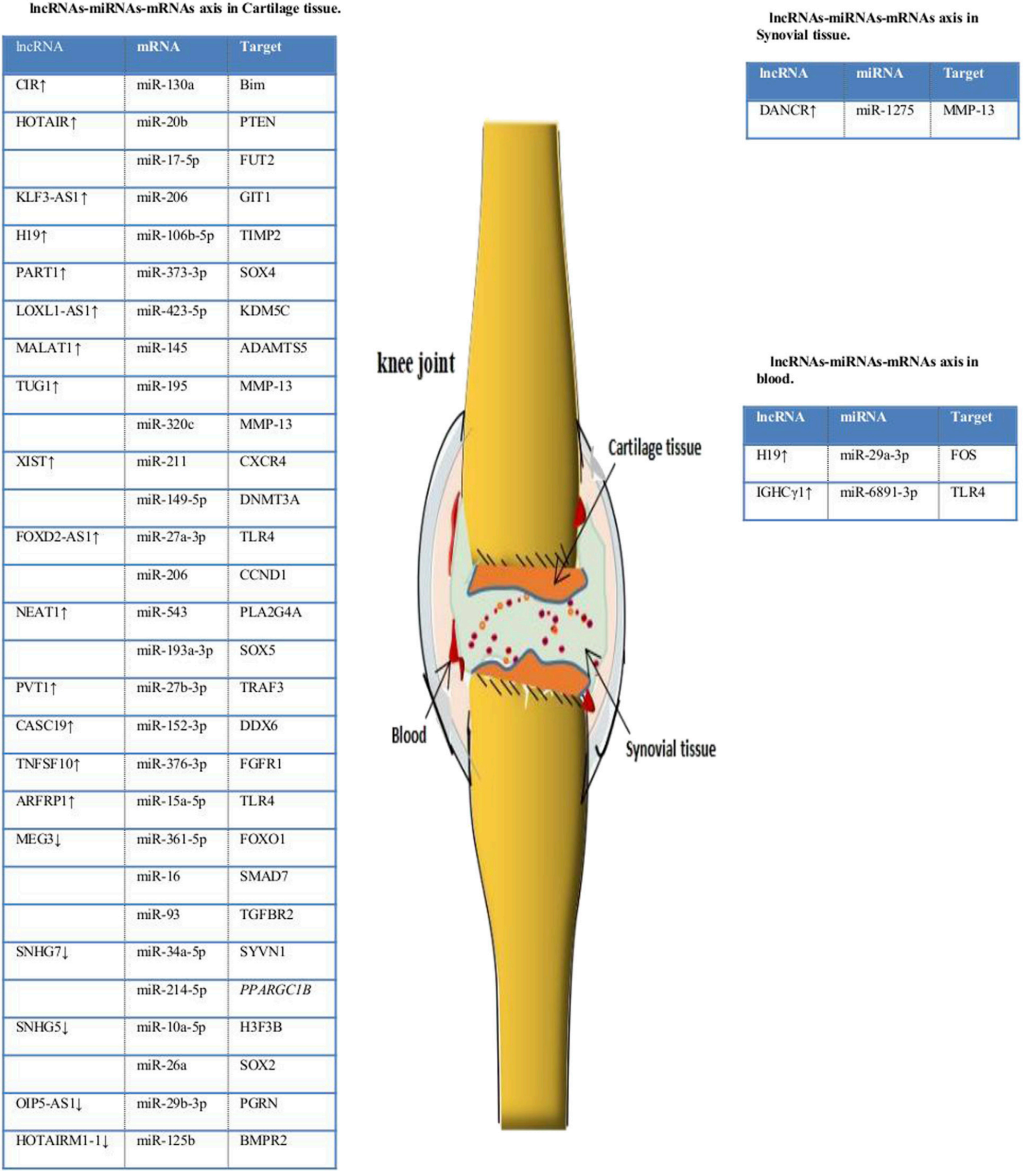
lncRNA–miRNA–mRNA axis in OA. lncRNA can combine with miRNA to promote the expression of related target genes. PTEN = phosphatase and tensin homolog; FUT2 = fucosyltransferase 2; Timp2 = tissue inhibitor of metalloproteinase 2; KDM5C = lysine demethylase 5C; DNMT3A = DNA methyltransferase 3A; TLR4 = toll-like receptor 4; CCND1 = cyclin D1; KLF4 = Krüppel-like factor 4; SYVN1 = synoviolin 1; PPARGC1B = PPARG coactivator 1 beta; H3F3B = H3 histone family 3B; PGRN = progranulin; DNM3OS = dynamin 3 opposite strand; BMPR2 = bone morphogenetic protein receptor 2.

Studies have shown that lncRNA–miRNA–mRNA axis plays a vital control effect in the progression of several diseases, such as cardiovascular disease and cancer (He et al., 2018; Wang et al., 2019c). The mechanisms of interaction of lncRNAs, miRNAs, and mRNAs in various diseases are as follows: 1) The structure of most lncRNAs is similar to mRNAs, and miRNAs binding to mRNAs can reduce the expression of lncRNAs. lncRNA and miRNA compete to bind the 3′-UTR of target gene mRNA, thereby indirectly inhibiting the interaction between miRNA and mRNA. For example, in Alzheimer’s disease, the post-transcriptional regulation of BACE1 involves miR-485-5p, and the specific antisense transcription of BACE1 forms lncRNA-BacE1-As, which compete with lncRNA-Bace1-As to bind to the binding sites of related mRNAs (Faghihi et al., 2010). 2) lncRNAs sponge miRNAs as competitive endogenous RNAs (ceRNAs). lncRNA molecules contain miRNA binding sites, which can bind to miRNA, inhibit the interaction between miRNA and mRNA, improve the expression level of related mRNA, and regulate the expression of target genes. For example, Zhang et al. (2020l) constructed a complete mRNA-LncRNA-miRNA ceRNA regulatory network; lncRNAs ENST00000326237.3, ENST00000399702.5, and ENST00000463727.1 were found to regulate related genes through competitive binding of the same miRNA has-miR-1260a. Kong et al. (2019) demonstrated that lncRNA—CDC6 can further regulate CDC6 expression through direct uptake of miR-215 as a ceRNA. Luan et al.(Luan and Wang, 2018) found that in cervical cancer, XLOC_006390 may act as ceRNA and bind with miR-331-3p and miR-338-3p, thus regulating the expression of genes related to cervical cancer. 3) miRNAs mediate the degradation of lncRNAs. For example, miRNA-150 is the target gene for lncRNA CASC11 in human plasma, and increased concentrations of miRNA-150 decrease the activity of lncRNA CASC11(Luo et al., 2019b). 4) lncRNAs act as miRNAs precursors. For example, Tao et al. (2017) found that miR-869a and miR-160c could be clipped from lncRNAs npc83 and npc521. However, in OA, lncRNA mainly binds to miRNA as a competitive endogenous RNA (ceRNA), inhibiting its target genes’ expression and regulating OA’s progression by regulating cell proliferation, apoptosis, autophagy and extracellular matrix (ECM) degradation (Figure 4).

There are many examples where lncRNA functions as a binding of ceRNA to miRNA in OA. For example, Zhang et al. (2020m) took IL -1β-induced OA chondrocytes as the research object to study the molecular mechanism of LINC00511 in regulating OA. The study found that the expression of LINC00511 was up-regulated, and the lncRNA could be used as a sponge of miR-150-5p and combined with 3′-UTR of transcription factor inhibit the proliferation of chondrocytes, promote apoptosis and degradation of ECM, and finally regulate OA. Liu et al. (2018) established an OA chondrocyte model induced by IL -1β and an OA mouse model caused by collagenase. The experiments were performed *in vivo* and *in vitro* at two levels, and the cell state was examined by the CCK-8 method and flow cytometry. Studies have found that KLF3-AS1, as a ceRNA interacting with miR-206, promotes the expression of GIT1 and then promotes the proliferation of chondrocytes and inhibits apoptosis, ultimately alleviating the progression of OA. Likewise, Tian et al. (2020) studied the relationship between SNHG7, miR-34a-5p, and SYVN1 in human chondrocytes. It has been found that in OA tissues, SNHG7 is down-regulated, and SNHG7 can regulate SYVN1 by sponging miR-34a-5p, thereby promoting cell proliferation and inhibiting apoptosis and autophagy. In addition, studies have found that lncRNA XIST is up-regulated in OA articular cartilage. Like a sponge, XIST regulates the target proteins miR-211, miR-17-5p, miR-149-5p, and miR-27b-3p, thereby promoting the proliferation and apoptosis of chondrocytes and finally inducing OA (Li et al., 2018b; Zhu et al., 2021). These results suggest that lncRNAs can act as miRNA sponges in the interaction of lncRNAs, miRNAs, and mRNA in OA.

### Interactions Between circRNAs, miRNAs and mRNAs in OA

**FIGURE 5:**
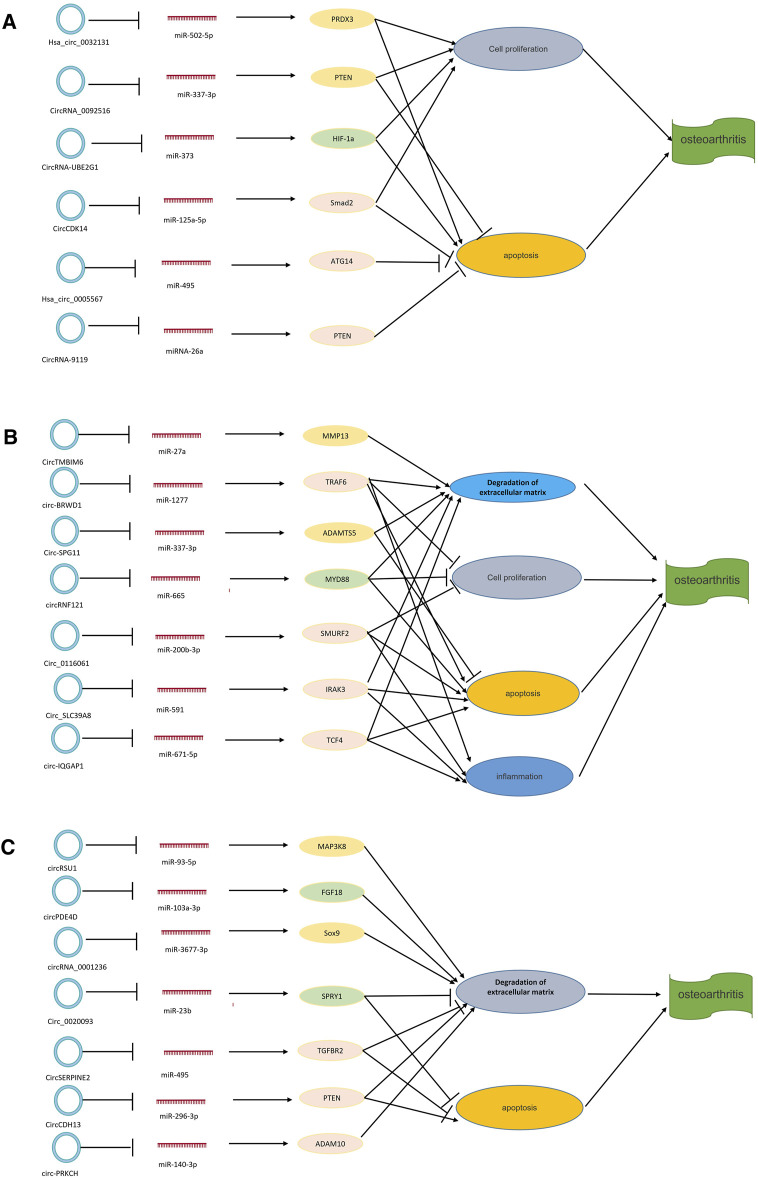
circRNA–miRNA–mRNA axis in OA. circRNAs can combine with miRNAs to promote the expression of related target genes. **(A)** circRNAs that play a role in cell proliferation and apoptosis. **(B)** circRNAs that play a role in degradation of the extracellular matrix and apoptosis. **(C)** circRNAs that play a role in degradation of the extracellular matrix, cell proliferation, apoptosis, and inflammation. NAMPT = nicotinamide phosphoribosyltransferase; MMP13 = matrix metalloproteinase.

Currently, research on the mechanism of interactions between circRNAs, miRNAs, and mRNAs is growing (Peng et al., 2020). circRNAs and miRNAs are closely related to the expression of disease-related mRNAs, and interactions between circRNAs, miRNAs, and mRNAs may be involved in the pathological mechanism of OA (Figure 5). At present, research on the interaction mechanism of circRNAs, miRNAs, and mRNAs is not comprehensive. Relevant research has three main types: 1) circRNAs interact with miRNAs. miRNA interacts with mRNA to inhibit mRNA expression. circRNA molecules contain miRNA binding sites, which can sponge miRNA and release miRNA’s inhibitory effect on target genes. For example, Hansen et al. (2013) found that CiRS-7 could sponge miR-7, inhibit the binding of miR-7 and its target genes, and indirectly promote the expression of related mRNA. Other research suggests that hsa_circ_101237, like a sponge for miRNA490-3p, promotes the expression of its target gene MAPK1. In patients with lung cancer, hsa_circ_101237 expression is up-regulated, thereby promoting the proliferation, differentiation, and migration of lung cancer cells (Zhang et al., 2020o). 2) circRNA can regulate the splicing of pre-mRNA, thus affecting the production of protein. 3) circRNA can pair with targeted mRNA directly through local bases. As the circRNA molecule is rich in miRNA binding sites, the circ RNA molecule functions as a miRNA sponge in cells so that the inhibition effect of the miRNA on target genes can be released, and the expression level of the target genes is increased. Therefore, in OA, the interaction mechanism of circRNA, miRNA, and mRNA is mainly circRNA sponging miRNA (Kulcheski et al., 2016). Many circRNA expressions in OA have been changed, and OA is regulated by adsorbing a specific miRNA. For example, hsa_circ_0005567 is down-regulated in OA patients and, by competitively binding to miR-495, terminates Atg14 expression and eventually induces human chondrocyte apoptosis (Zhang et al., 2020f); hsa_circ_0032131 is up-regulated in the human body, and knocking out hsa_circ_0032131 inactivates the STAT3 signaling pathway by sponging miR-502-5p, thereby relieving symptoms of OA in the body (Xu and Ma, 2021); circPSM3 is up-regulated in OA chondrocytes, and its low expression promotes chondrogenesis and OA development. circPSM3 can inhibit OA chondrogenesis by sponging miRNA-296-5p (Ni et al., 2020a). All these results prove the mechanism of circRNA sponge miRNA in osteoarthritis.

Other studies have found interactions between circRNA, miRNA, and mRNA. Shen et al. (2020a) established a rabbit model of OA and studied the role and mechanism of circCDK14 in OA by quantitative reverse transcriptase-polymerase chain reaction (RT-PCR) and other methods. miR-125a-5p is a downstream target protein of circCDK14, while Smad2 is an mRNA target protein of circCDK14. The mechanism of action of circCDK14 in OA is to down-regulate the expression of Smad2 through the sponge action of miR-125a-5p, resulting in dysfunction of the TGF-β signaling pathway. Chen et al. (2020a) studied the expression and action mechanism of circRNA-9119 in OA patients using bioinformatics prediction and double luciferase reporter gene detection. They found that the expression of circRNA-9119 was down-regulated to provide a sponge effect on miR-26a. At the same time, miR-26a targeted the 3' -UTR of PTEN to promote cell proliferation and inhibit apoptosis. Their results all demonstrated the mechanism of the interaction between circRNAs, miRNAs, and mRNAs in OA.

## Clinical Implications

At present, the incidence of OA is very high, and its pathogenesis is still unclear. Studying the specific pathological process and molecular pathway of OA is of great clinical significance (Duan et al., 2020). First, ncRNA can be used to diagnose OA. The expression of many ncRNAs between patients with OA and normal individuals have remarkable differences, which can be seen in humans and animals. For example, Huang et al. (2019c) showed that miRNA-204 and miRNA-211 are decreased in OA, resulting in Runx2 accumulation in multiple types of joint cells and elevated OA markers, and leading to total joint degeneration. Second, several ncRNAs are associated with the prognosis of OA. Rousseau et al. (2020) took the miRNAs in the serum of female patients with KOA as the research objects. He first made a preliminary screening of the research objects through next-generation sequencing and then further analyzed the research objects through RT-QPCR. He found that miR-146A-5p is up-regulated in patients with mild OA, and the prognosis of OA caused by the up-regulation of miRNA is relatively good. In addition, the increase of miR-186-5p in an individual means that the individual might have the imaging changes of OA in the past 4 years, which could be prevented in advance to avoid the occurrence of OA as much as possible. Finally, several ncRNAs can be used for the treatment of OA. Several new drugs can be developed to promote or inhibit several ncRNAs, or change the pathway of action of ncRNA to treat OA. For example, miR-93 is down-regulated in mice with OA and lipopolysaccharide-treated chondrocytes, and acts directly on TLR4 to exert biological effects. miR-93 regulates OA by inhibiting the TLR4/NF-κB pathway, lipopolysaccharide-induced inflammation, and apoptosis. In patients with OA and down-regulation of miR-93, corresponding drugs can be developed to promote its up-regulation and inhibit the aggravation of OA (Ding et al., 2019). These studies indicate that ncRNA has great potential for clinical use in OA. At present, most of the tissue comes from cartilage and is found in the knee joint, and the chondrocytes are cultured to construct the OA cell model. Further research is needed, and more clinical trials must be explored to find biomarkers associated with OA while developing the immense potential of ncRNA.

## Conclusion

In recent years, ncRNAs have become one of the most widely studied fields in the development of OA. However, the studies on the regulation of miRNA, lncRNA, and circRNA in diseases and their use as indicators for diagnosis or treatment of OA are still in the early stages, and the mechanism of action ofOA, which may involve multiple signaling pathways, is still unclear. This study reviews theinteractions between lncRNA/circRNA and miRNA in OA. Through high-throughput sequencingtechnologies such as microarray analysis and RNA sequencing, the findings reveal that a large number of miRNA, lncRNA, and circRNA are dysregulated in patients with OA, and the clinical trials related to ncRNA and OA are summarized. The present research progress of ncRNA in the prevention, diagnosis, and treatment of OA is illustrated, which provides a basis for the treatment of OA by ncRNA in the future.
